# Oncolytic virotherapy counteracts the selection of IFN-unresponsive cancer cells post-immunotherapy but is limited by the emergence of dedifferentiated cancer cells

**DOI:** 10.3389/fimmu.2026.1875225

**Published:** 2026-06-22

**Authors:** Susan Gellert, Bastian Kruse, Johannes Peters, Susanne Bonifatius, Thomas Tüting, Anthony C. Buzzai

**Affiliations:** 1 Laboratory of Experimental Dermatology, Department of Dermatology, University Hospital and Health Campus Immunology Infectiology and Inflammation (GC-I3), Otto-von-Guericke-University, Magdeburg, Germany; 2 Department of Experimental Immunology, Institute of Immunology, Eberhard Karls University of Tübingen, Tübingen, Germany; 3 M3 Research Center, University Hospital Tübingen, University of Tübingen, Tübingen, Germany; 4 The Kids Research Institute Australia, Nedlands, WA, Australia

**Keywords:** immune checkpoint inhibition, interferon, melanoma dedifferentiation, oncolytic virotherapy, therapy resistance

## Abstract

Genetic mutations disrupting interferon (IFN) signaling have been described as an evolutionary route for tumor cells to evade immune checkpoint inhibitor (ICI) therapy. We hypothesized that loss of IFN signaling not only enables cancer cells to evade immune surveillance, but also renders them susceptible to oncolytic viruses. To experimentally address this hypothesis, we generated IFN-unresponsive mouse melanoma cell variants via CRISPR/Cas9 and investigated the impact of sequential immuno-virotherapy on their evolutionary dynamics in immunocompetent syngeneic hosts. In wild-type mice, Natural Killer (NK) cells eliminated IFN-unresponsive subclones in mixed tumor cell transplants due to their very low expression of major histocompatibility complex class I (MHC-I) molecules. Upon NK cell depletion, IFN-unresponsive subclones established and were preferentially selected by T cell-directed immunotherapy, consistent with observations in patients. Sequential oncolytic virotherapy was able to specifically target and counter-select these IFN-unresponsive clones. Unexpectedly, IFN-responsive tumor cells re-emerged with a dedifferentiated phenotype that resisted both immune and viral control. Together, these findings touch upon the paradoxical anti- and pro-tumoral roles of cancer cell-intrinsic IFN signaling and highlight dedifferentiation as a potential convergent resistance mechanism that ultimately limits the efficacy of both treatment approaches. Our findings may provide an explanation for why combination immunotherapy and oncolytic virotherapy did not meet clinical expectations.

## Introduction

The recent success of immunotherapy has transformed the landscape of cancer treatment. Immune checkpoint inhibitors (ICI) and adoptive cell transfer (ACT) have led to improved survival and even durable responses in patients with various cancers, including melanoma (1–3). However, therapeutic resistance remains a major barrier, with many tumors demonstrating intrinsic or acquired resistance to these approaches (4). To enhance the clinical efficacy of immunotherapies and broaden the cohort of responsive patients, rationally designed combination strategies are essential. One such approach involves the integration of ICIs with oncolytic viruses, which has been shown to potentiate anti-tumor immunity by augmenting T cell infiltration and activation within the tumor microenvironment, particularly against melanoma (5).

The interferon (IFN) signaling axis plays a pivotal role in shaping both immunotherapy and virotherapy outcomes by orchestrating innate immune activation, enhancing antigen presentation, and modulating the tumor microenvironment to favor cytotoxic T cell infiltration and function. Clinical transcriptomic studies show that IFN signatures in tumors strongly correlate with T cell infiltration (6) and response to PD-1-based therapies (7). Clustered Regularly Interspaced Short Palindromic Repeats (CRISPR)-based genetic screens demonstrated the importance of cancer cell intrinsic IFN signaling for promoting anti-tumor immune responses (8). In line with this, the emergence of tumor cell variants with genetic loss of IFN signaling pathway components has been linked to ICI therapy resistance in clinical settings (9, 10).

The duality of IFN signaling, acting as both a driver of immune-mediated tumor rejection and a barrier to viral replication, has profound implications for oncolytic virotherapy. Oncolytic viruses were initially administered to lyse tumor cells and stimulate immune responses by activating type I IFNs. This was anticipated to transform immunologically “cold” tumors, lacking immune infiltration, into “hot” tumors with enhanced antigen presentation and T cell recruitment (11). However, IFN signaling also restricts viral infection, limiting therapeutic efficacy. Therefore, more recent studies have focused on the potential ability of oncolytic viruses to preferentially target and lyse IFN signaling-deficient tumor cells *in vitro* (12, 13), that emerge during resistance.

Herein, we investigated the hypothesis that oncolytic viruses are able to selectively target and eliminate IFN signaling-deficient tumor cells that emerge *in vivo* following immunotherapy. We established an experimental setting that allowed us to explore the evolutionary dynamics of IFN-unresponsive subclones in transplanted mouse melanomas. We first generated IFN-signaling deficient mouse melanoma cells by disrupting the Janus Kinase 1 (Jak1) gene using CRISPR/Cas9. Jak1-deficient tumor cells exhibited a complete loss of responsiveness to IFNs. We found that this loss of IFN-signaling impaired major histocompatibility complex class I (MHC-I) upregulation, rendering tumor cells susceptible to Natural Killer (NK) cell-mediated killing *in vitro.* In subsequent work *in vivo*, we found that IFN-unresponsive subclones were eliminated by NK cells in transplanted mouse melanomas, consistent with our observations *in vitro*. In mice depleted of NK cells, both ICI and ACT immunotherapies led to the selective emergence of IFN-unresponsive tumor subclones from mixed populations. Importantly, subsequent oncolytic virotherapy following immunotherapy was able to selectively target and eliminate these resistant IFN signaling-deficient subpopulations. Surprisingly, following sequential immuno-virotherapy, IFN-responsive tumor cells re-emerged with a dedifferentiated phenotype that resisted both T cell control and viral oncolysis. Together, these findings touch upon the paradoxical anti- and pro-tumoral roles of cancer cell-intrinsic IFN signaling and highlight dedifferentiation as a potential convergent resistance mechanism that ultimately limits the efficacy of both treatment approaches.

## Methods

### Mice

Mice were housed in temperature and humidity-controlled environment on a 12 h light/dark cycle. Wild type C57BL/6J (RRID: IMSR_JAX:000664) mice were purchased from Janvier (Le Genest- Saint-Isle, France). T cell receptor-transgenic Pmel-1 (B6.Cg-Thy1a/Cy Tg(TcraTcrb)8Rest/J; RRID: IMSR_JAX:005023) were originally purchased from Jackson Laboratories and were bred in house. Age matched cohorts of tumor developing mice were randomly allocated to the different experimental groups. All animal experiments were conducted with male mice on the C57BL/6J background under specific pathogen-free conditions in individually ventilated cages according to the institutional and national guidelines for the care and use of laboratory animals with approval by the Ethics Committee of the Office for Veterinary Affairs of the State of Saxony-Anhalt, Germany (permit license numbers 42502-2–1393 Uni MD, 42502-2–1586 Uni MD, 42502-2–1615 Uni MD, 42502-2–1672 Uni MD) in accordance with legislation of both the European Union (Council Directive 499 2010/63/EU) and the Federal Republic of Germany (according to § 8, Section 1 TierSchG, and TierSchVersV).

### Cell lines and cell culture

The HCmel12 mouse melanoma cell line was derived from a primary melanoma of an Hgf-Cdk4^R24C^ mouse in our laboratory as described previously (6). HCmel12 cells were cultured in RPMI 1640 medium (Life Technologies, Carlsbad, CA) supplemented with 10% fetal bovine serum (FCS, Biochrome, Berlin, Germany), 1 mM Sodium Pyruvate (Gibco), 2 mM L-glutamine, 10 mM non-essential amino acids, 1 mM HEPES, 100 U/mL penicillin and 100 μg/mL streptomycin (all from Life Technologies) and 20 μM 2-mercaptoethanol (Sigma, St. Louis, MO). All cell lines were cultured in a humidified incubator with 5% CO_2_ at 37 °C. For cytokine stimulations, cells were treated with indicated concentrations of mouse IFNg (R&D Systems). Monoclonal *Jak1* knockout variants of HCmel12, were generated using CRISPR-Cas9 genome engineering. After characterizing the clones first individually, experiments were carried out using mixtures of four monoclones. For validation experiments, tumor cells were treated with 1000 U/ml of recombinant IFNg (Peprotech) for 72 h. All cell lines used in our study were routinely tested to be mycoplasma free.

### CRISPR/Cas9-mediated cell engineering and validation

To generate HCmel12 Jak1 KO cell lines, specific sgRNAs were first designed using CRISPOR tool (http://crispor.tefor.net/, RRID: SCR_015935). Subconfluent target cell cultures were then transfected with the Px458 (pSpCas9(BB)-2A-GFP, RRID: Addgene_48138) plasmid, which expressed an eGFP marker gene in addition to the subcloned sgRNA of interest (or empty plasmid as control). Fugene HD transfection reagent (Promega, Fitchburg, WI) was used according to the manufacturer’s instructions for the transfections. The next day, the transfected cells were single cell sorted into flat bottom 96-well plate wells with FACSAria III Cell Sorter (BD- Biosciences, San Jose, CA). The cell cultures were expanded, genomic DNA isolated with NucleoSpin Tissue kit (Macherey&Nagel) and barcoded using a two-step polymerase chain reaction (PCR) protocol as described previously (14). NGS was carried out with MiSeq Gene and Small Genome Sequencer (Illumina, RRID: SCR_016379). For the detection of insertions or deletions, the Outknocker program (http://www.outknocker.org/) was utilized as previously described (15).

### NK cell-mediated killing assay

To assess the ability of NK cells to kill HCmel12 melanoma cells, the CRISPR ctrl and Jak1-KO cell lines were co-cultured with NK cells. To this end, 10^4^ HCmel12 CRISPR ctrl or Jak1-KO cells were co-cultured with 10^5^ MACS purified NK cells. Four hours later, cell death of the tumor cells was measured using the FITC Annexin V Apoptosis Detection Kit I (BD Pharmingen) and analyzed via flow cytometry.

### Oncolytic virus (SFV-VA7-eGFP) preparation

SFV−VA7−eGFP is a replication−competent alphavirus vector derived from the attenuated Semliki Forest virus (SFV) strain A7(74) (16). The virus replicates efficiently in infected cells, leading to rapid cytolysis, and shows preferential replication in tumor cells compared to non−malignant cells (17). Replication is highly sensitive to type I IFN signaling, such that IFN−competent cells restrict viral spread, whereas IFN−deficient cells remain permissive (18). Virus stocks were generated by *in vitro* transcription of the SFV−VA7 genome followed by transfection into BHK−21 cells. Supernatants containing primary virus were harvested 24-48 h post−transfection and further amplified by infecting confluent BHK−21 cultures. Virus-containing supernatants were clarified by centrifugation, filtered (0.2 µm), aliquoted, and stored at -70 °C until use. Virus titers were determined by plaque assay on Vero(B) cells using standard serial dilution and agarose overlay procedures to quantify plaque-forming units (PFU).

### Tumor transplantations

For tumor transplantations, 2x10^5^ tumor cells suspended in 50 µL volume of PBS and were injected intracutaneously (i.c.) to the right flanks of the mice, using a 30G (0.3 x 13 mm) injection needle (BD). Tumor growth was monitored three times per week by measuring their average diameter using a Caliper instrument. The mice were sacrificed when tumors reached the end-point size (max. 15 mm) or showed signs of illness in accordance with local ethics regulations. In indicated experiments, NK cells were depleted one day before tumor transplantations by injecting 200 µg of anti-NK1.1 (PK136, BioXcell, RRID: AB_1107737) to allow growth of HCmel12 Jak1-KO tumor cells. InVivoMab mouse IgG2 (BioXcell, RRID: AB_1107771) was used as the isotype control antibody for NK cell depletion. The depletion efficacy was verified via flow cytometry from the blood of the mice.

### Immune therapy treatment

For dual ICI experiments, 200 µg of anti-CTLA4 (9H10, BioXcell, RRID: AB_10950184) and 250 µg of anti-PD1 (RMP1-14, BioXcell, RRID: AB_10949053) antibodies diluted in InVivoPure pH 7.0 Dilution Buffer were injected twice weekly for two weeks intraperitoneally (i.p.) when tumors reached a mean diameter of 3–5 mm. The adoptive cell transfer (ACT) protocol of Pmel-1 CD8+ T cells has been established in the laboratory and was carried out as previously described (14, 19). Once transplanted tumors reached 3–5 mm, mice were preconditioned by a single i.p. injection of 2 mg of cyclophosphamide in 100 µl PBS one day before the intravenous (i.v.) transfer of 5x10^5^ Pmel-1/gp100 specific CD8+ T cells in 100 µl PBS. The transferred T cells were stimulated *in vivo* by a single i.p. injection of the recombinant adenoviral vaccine Ad-PT in 100 µl of PBS (2.5 x10^7^ PFU). On day 3, 6 and 9 after the transfer, tumors were injected with 100 µl of 50 µg of CpG 1826 (MWG Biotech) and 50 µg of polyinosinic:polycytidylic acid (polyI:C, Invivogen). Seven days after the T cell transfer, blood of mice was taken to validate expansion of transferred CD8+ T cells by flow cytometry. Ten days following the immunotherapy treatments, 10^6^ PFU of sucrose-purified SFV-VA7-eGFP was injected intratumorally.

### Flow cytometry

Staining of single cell suspensions was performed according to standard protocols. *In vitro* cell cultures after cytokine stimulation were incubated with H2kb-PE (1:500) (Biolegend, RRID: AB_313734). To confirm NK cell depletion in mice, blood harvested from mice was lysed using the RBC lysis buffer (Biolegend) and was subsequently stained with NKp46-APC (1:100, Biolegend, RRID: AB_10612749), CD45-FITC (1:1, 000; Biolegend, RRID: AB_2563541) and CD3-PE (1:200; BD, RRID: AB_394597). To confirm CD8+ T cell expansion, blood harvested from mice 7 days post-adoptive transfer and was lysed using the RBC lysis buffer (Biolegend), then subsequently stained with CD3-BV421 (1:400, Biolegend, RRID: AB_2562553), CD45-APC Fire 750 (1:1, 600, Biolegend, RRID: AB_2750440), CD90.1-PerCP (1:100, BD, RRID: AB_396611) and CD8-PE (1:800; BD, RRID: AB_394571). To determine the percentage of mCherry+ HCmel12 Jak1-KO cells, tumors were homogenized into single cell suspensions. All data was acquired analyzed using an Attune Nxt Flow Cytometer (ThermoFisher Scientific, RRID: SCR_019590). Gating and analyses were performed with the FlowJo v.10.8.1 Software (Tree Star Inc., RRID: SCR_008520). Fluorescence-activated cell sorting was performed using an Aria III (BD Biosciences).

### Immunohistochemistry

Mouse tumors were fixed overnight in formalin-free zinc fixative, embedded in paraffin, and sectioned (6 µm). Deparaffinised and rehydrated sections were subjected to heat-induced antigen retrieval in citrate buffer (pH 6.0). Sections were then incubated with primary antibodies against mouse gp100 (rabbit polyclonal, NBP1-69571, Novus Biologicals, RRID: AB_11023072) or SFV (rabbit polyclonal, kind gift from A. Hinkkanen), followed by anti-rabbit secondary antibodies (Jackson ImmunoResearch). Detection was performed using the ultraView Universal Alkaline Phosphatase Red Detection Kit (Ventana, Oro Valley, Arizona, USA), with hematoxylin counterstaining. Stained sections were imaged with an Axio Imager A1 light microscope (ZEISS, Oberkochen, Germany) and digitized with a NanoZoomer-SQ digital slide scanner (Hamamatsu Photonics, Hamamatsu, Japan). IHC staining was assessed qualitatively, and representative images are shown.

### Real-time quantitative PCR

Total RNA from subconfluent cell cultures was isolated using NucleoSpin RNA XS kit (Macherey-Nagel, Düren, Germany) and RNA concentration was quantified with a Spark 10 M multimode microplate reader (Tecan Group; RRID: SCR_021897). Complementary DNA was synthesized with GoScript reverse transcription kit with oligo(dT)15 primers (Promega) according to the manufacturer’s instructions. Real-time quantitative PCR analyses were carried out as technical duplicates using GoTaq quantitative PCR Master Mix (Promega) and peqSTAR 96Q-thermocycler (PEQLAB Biotechnology, Erlangen, Germany). Relative expression in comparison to the reference gene ubiquitin was calculated using the equation: 2^-ΔCt^*1000, where ΔCt = Ct(target gene) − Ct(reference gene).

### Statistical tests

All statistical tests for *in vitro* and *in vivo* experiments were performed using GraphPad Prism 8 (RRID: SCR_002798). The used tests are specified in the figure legends.

### Data availability

The data supporting the findings are available upon request.

## Results

### IFN-unresponsive melanoma cells show impaired MHC-I upregulation and are subject to NK cell immunosurveillance

**Figure 1:**
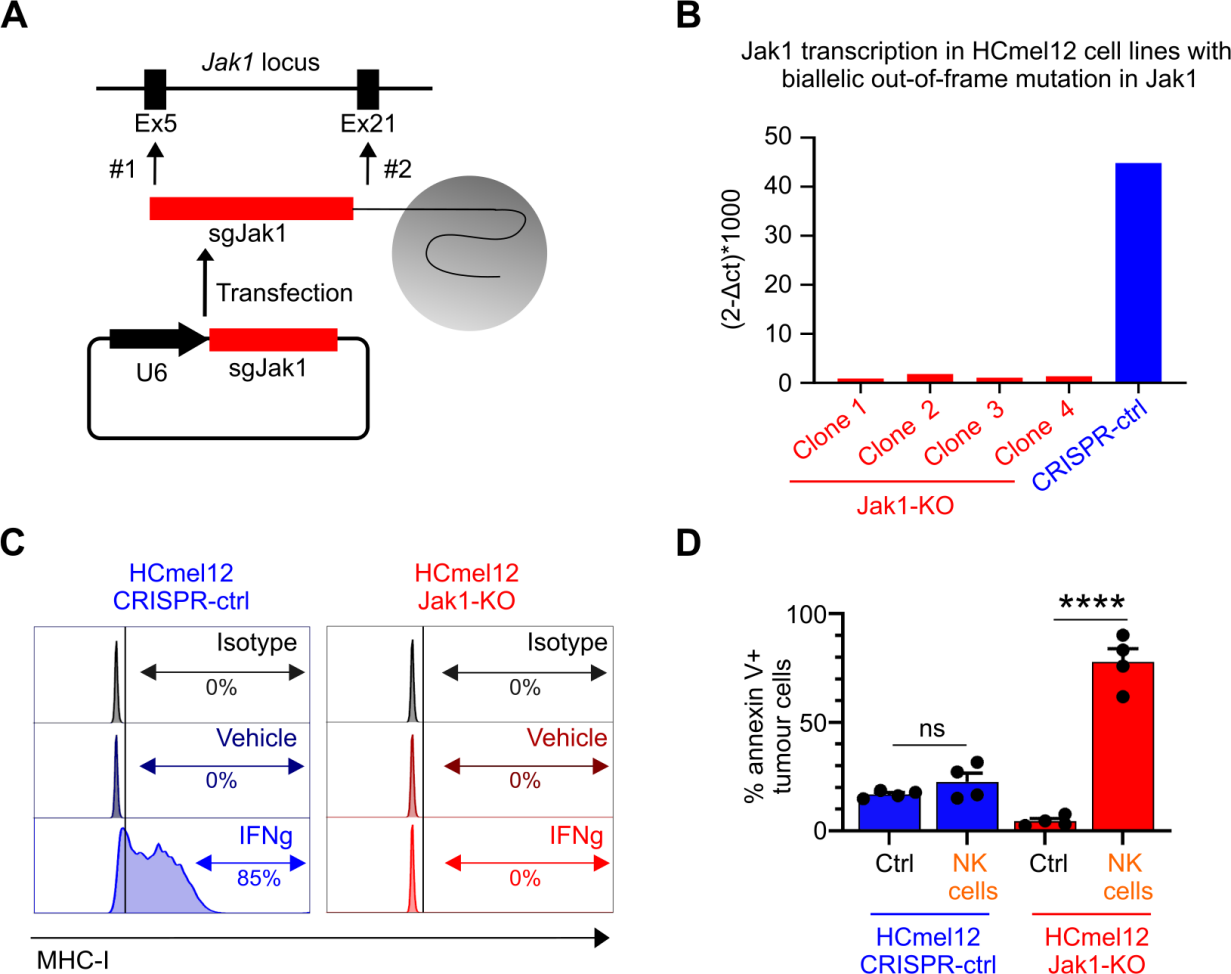
Genetically IFN signaling-deficient melanoma cells show impaired MHC upregulation and are subject to NK cell-mediated killing *in vitro.* **(A)** Graphical representation of CRISPR-mediated targeting of the Jak1 gene. **(B)** mRNA levels of Jak1 in the four HCmel12 Jak1-KO cell lines (red) and the HCmel12 CRISPR-ctrl cell line (blue). **(C)** Flow cytometry histograms of the MHC-I expression in the HCmel12 CRISPR-ctrl cell line (left) and the HCmel12 Jak1-KO cell line (right) either treated with IFNg (bottom) or left untreated (middle). Isotype stain is shown as control (top). **(D)** Percentage of Annexin V+ HCmel12 CRISPR-ctrl cells (left) or HCmel12 Jak1-KO tumor cells (right) after 4 h co-culture with NK cells. Statistical significance determined by Kruskal–Wallis test with Dunn’s multiple comparison test (ns, non-significant and ****p<0.001).

To experimentally model the impact of sequential immuno-virotherapy on the evolution dynamics of IFN-signaling deficient melanoma subclones, we generated IFN-unresponsive HCmel12 Jak1-KO mouse melanoma cells using CRISPR/Cas9 gene editing by targeting two separate exons of the Jak1 gene, a central mediator of the type I and type II IFN signaling pathways (**Figure 1a**). Following gene editing, we expanded monoclones and tested for disruption of the Jak1 gene by next generation sequencing of the targeted DNA sequence. Monoclones with homozygous out-of-frame mutations in the Jak1 gene were selected and the impact of gene editing validated at the transcriptional level by quantitative PCR (**Figure 1b**). Further experiments were performed using an equal mixture of four validated HCmel12 Jak1-KO monoclones, to avoid bias of a single clone.

**Figure 2:**
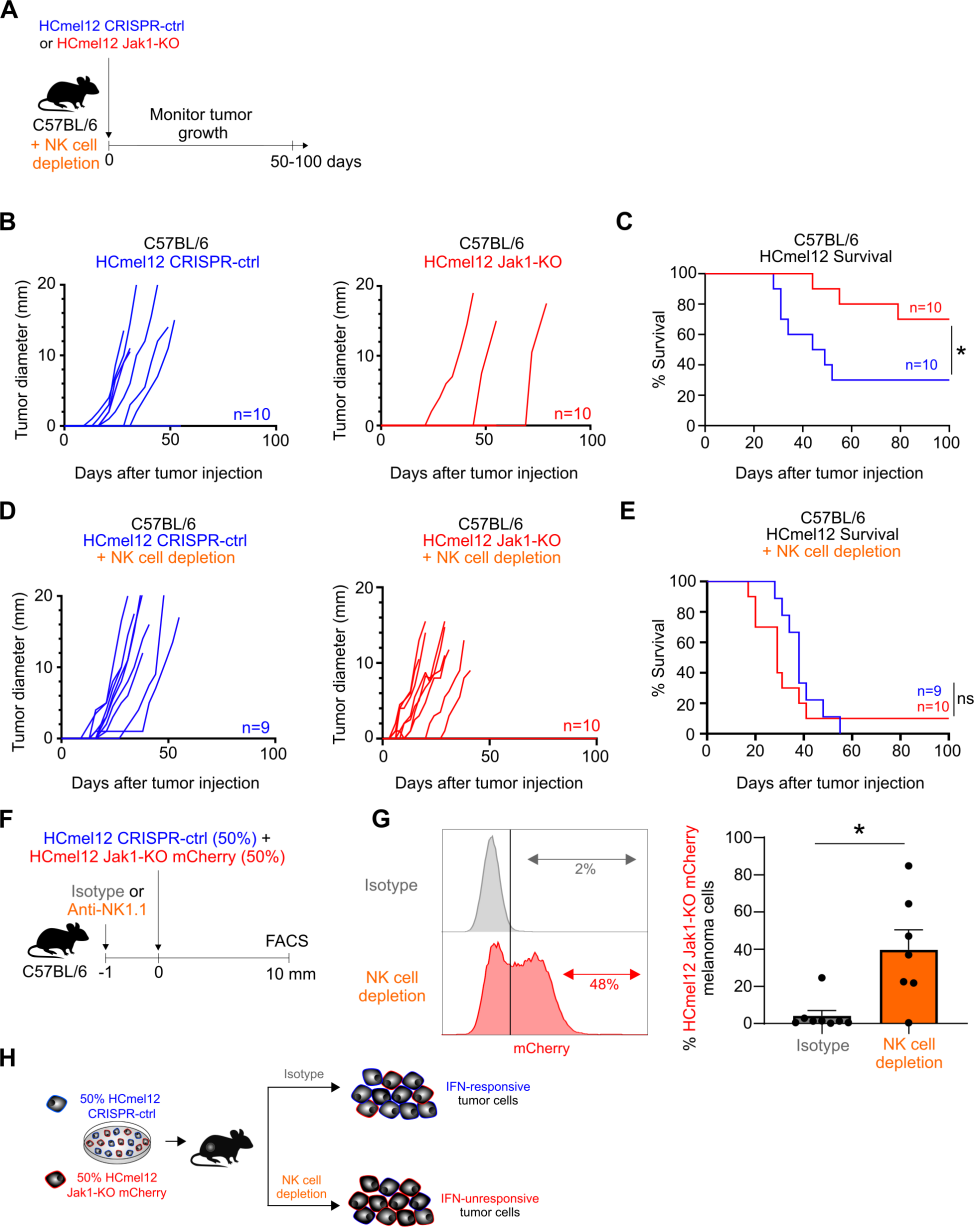
Genetically IFN signaling-deficient melanoma cells are subject to NK cell immunosurveillance *in vivo*. **(A)** Graphical representation of *in vivo* experiment. C57BL/6 mice were either injected with 200 µg of the anti-NK1.1 antibody one day prior to the tumor injection or left untreated. Mice were then inoculated with HCmel12 CRISPR-ctrl or HCmel12 Jak1-KO tumor cells. **(B)** Individual tumor growth curves of C57BL/6 mice injected with HCmel12 CRISPR-ctrl (left) or HCmel12 Jak1-KO tumors (right). **(C)** Kaplan-Meier survival curve of C57BL/6 mice injected with HCmel12 CRISPR-ctrl or HCmel12 Jak1-KO tumors. Survival was statistically compared using a log-rank Mantel–Cox test (*p<0.05). **(D)** Individual tumor growth curves of NK cell-depleted C57BL/6 mice injected with HCmel12 CRISPR-ctrl (left) or HCmel12 Jak1-KO tumors (right). **(E)** Kaplan-Meier survival curve of NK cell-depleted C57BL/6 mice injected with HCmel12 CRISPR-ctrl or HCmel12 Jak1-KO tumors. Survival was statistically compared using a log-rank Mantel–Cox test (ns, non-significant). **(F)** Groups of C57BL/6 mice were either injected with 200 µg of anti-NK1.1 one day prior to the tumor injection or left untreated. Mice were then inoculated with a mixture of HCmel12 CRISPR-ctrl (50%) and HCmel12 Jak1-KO-mCherry (50%) mouse melanoma cells. **(G)** Left: Representative histograms showing the percentage of HCmel12 Jak1-KO-mCherry cells in progressively growing tumors of the indicated groups. Right: Data represented as mean ± SEM and compared statistically with two-tailed unpaired t-test (*p<0.05). **(H)** Graphical representation of the genetic evolution of melanoma cell subpopulations following NK cell depletion.

Functional validation of loss of IFN-responsiveness was confirmed by failure of the HCmel12 Jak1-KO to upregulate MHC-I expression following IFNg treatment (**Figure 1c**). Since loss of MHC-I renders cells susceptible to NK cell-mediated killing, we assessed the ability of NK cells to target IFN-unresponsive, MHC-I-deficient HCmel12 Jak1-KO cells. Therefore, we co-cultured HCmel12 CRISPR-ctrl and Jak1-KO cells with MACS-purified NK cells for 4 h and analyzed the induction of tumor cell death by flow cytometry using Annexin V staining. NK cells were not able to cause death of HCmel12 CRISPR-ctrl cells, but substantially increased death of HCmel12 Jak1-KO cells (**Figure 1d**). Next, we investigated the tumorigenicity and growth kinetics of IFN-unresponsive HCmel12 Jak1-KO tumor cells in direct comparison to their IFN-responsive HCmel12 CRISPR-ctrl cells. Tumor cells were injected into the flanks of wild-type C57BL/6 mice (**Figure 2a**). Tumors developed progressively in the majority of mice which received HCmel12 CRISPR-ctrl cells (**Figure 2b**). In contrast, the majority of mice implanted with HCmel12 Jak1-KO did not develop tumors, leading to increased survival (**Figure 2c**). This indicates that IFN-unresponsive tumors are much more immunogenic than their respective CRISPR-Ctrl counterparts in syngeneic immunocompetent mice. Therefore, we next performed the same experiment in mice depleted of NK cells prior to tumor transplantation. Strikingly, tumors developed in the majority of mice and no differences in tumor growth (**Figure 2d**) or survival (**Figure 2e**) was observed between mice inoculated with HCmel12 CRISPR-ctrl or HCmel12 Jak1-KO cells. Taken together, our results indicate that transplanted HCmel12 Jak1-KO melanoma cells were controlled by NK cell-mediated immune surveillance, likely since they do not constitutively express MHC-I molecules.

To assess whether HCmel12 Jak1-KO cells are selectively targeted by NK cells *in vivo*, we developed a model of heterogeneous tumors by inoculating mixtures of 50% IFN-unresponsive HCmel12 Jak1-KO melanoma cells expressing mCherry and 50% IFN-responsive HCmel12 CRISPR-ctrl melanoma cells into NK cell-depleted or non-depleted wild-type mice. When tumors were 10 mm in diameter, they were harvested and the proportion of mCherry-expressing cells was analyzed by flow cytometry (**Figure 2f**). Tumors from non-depleted mice were comprised of very few mCherry-expressing HCmel12 Jak1-KO melanoma cells, whereas tumors derived from NK-cell depleted mice contained significantly more mCherry+ HCmel12 Jak1-KO melanoma cells (**Figure 2g, h**).

### ICI immunotherapy selects for genetically IFN-unresponsive melanoma cells

**Figure 3:**
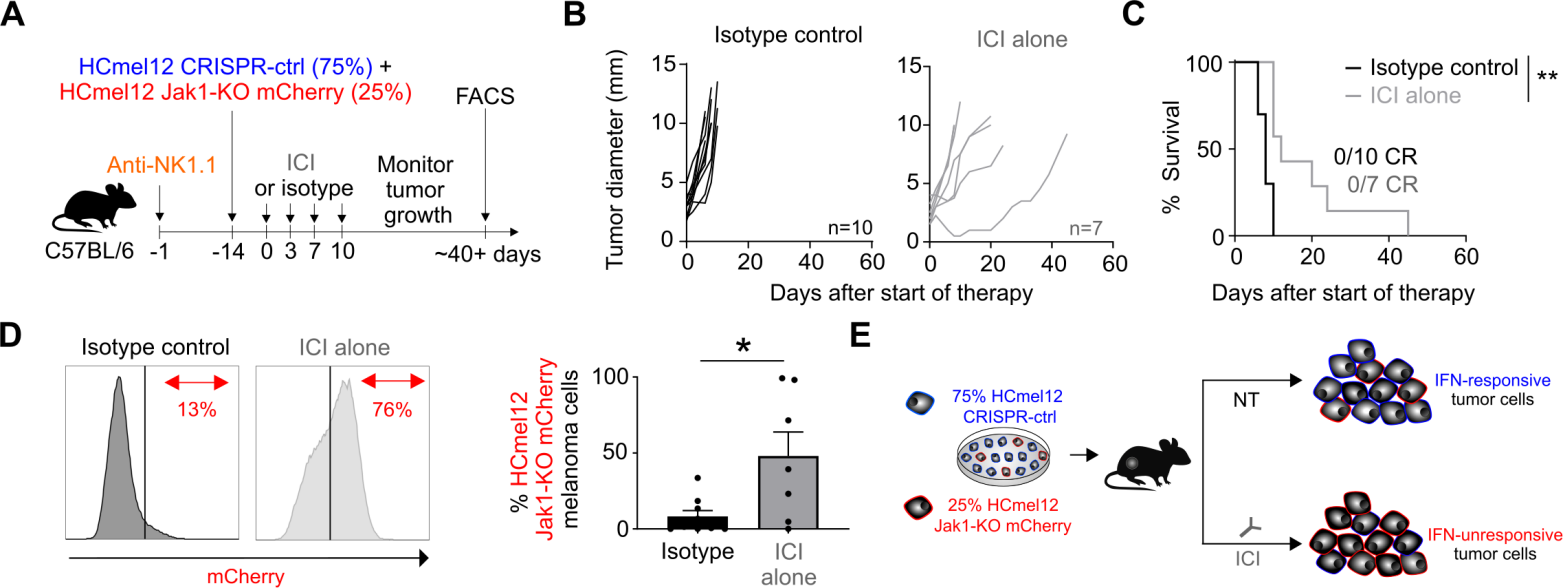
ICI immunotherapy selects for genetically IFN signaling-deficient melanoma cells. **(A)** Groups of C57BL/6 mice were either injected with 200 µg of the anti-NK1.1 antibody one day prior to the tumor injection or left untreated. Mice were then inoculated with a mixture of HCmel12 CRISPR-ctrl (75%) and HCmel12 Jak1-KO-mCherry (25%) mouse melanoma cells. Once established, tumors were treated with dual immune checkpoint inhibitor therapy (ICI: anti-CTLA-4 + anti-PD-1) twice weekly for two weeks. **(B)** Individual tumor growth curves in groups of mice treated as indicated. **(C)** Corresponding Kaplan-Meier survival graph (**p<0.005). **(D)** Left: Representative histograms showing the percentage of HCmel12 Jak1-KO-mCherry cells in progressively growing tumors of the indicated groups. Right: Data represented as mean ± SEM and compared statistically with two-tailed unpaired t-test (*p<0.05). **(E)** Graphical representation of the genetic evolution of melanoma cell subpopulations in response ICI.

The genetic selection of IFN-unresponsive melanoma cell subpopulations has been observed as a mechanism of resistance in patients treated with ICI (9). To better mimic the clinical scenario in an experimental setting where rare resistant clones are selected by ICI therapy, we generated heterogeneous tumors by inoculating mixtures of 25% IFN-unresponsive HCmel12 Jak1-KO melanoma cells expressing mCherry and 75% IFN-responsive HCmel12 CRISPR-ctrl melanoma cells into NK cell-depleted wild-type mice (**Figure 3a**). When tumors reached a size of 3–5 mm in diameter (~14 days), two groups of mice received intraperitoneal injections with anti-PD-1 and anti-CTLA4 mAbs (“ICI”). ICI therapy delayed tumor growth (**Figure 3b**) and prolonged survival when compared to the control group (**Figure 3c**). Flow cytometric analysis of escaping tumors showed that ICI therapy selected for IFN-unresponsive HCmel12 Jak1-KO-mCherry cells, consistent with clinical observations (**Figure 3d**). Therefore, immunotherapy selects for genetically IFN-signaling deficient melanoma subclones.

### Oncolytic virotherapy counteracts the selection of IFN-unresponsive melanoma cell subpopulations in heterogeneous tumors following ICI therapy

**Figure 4:**
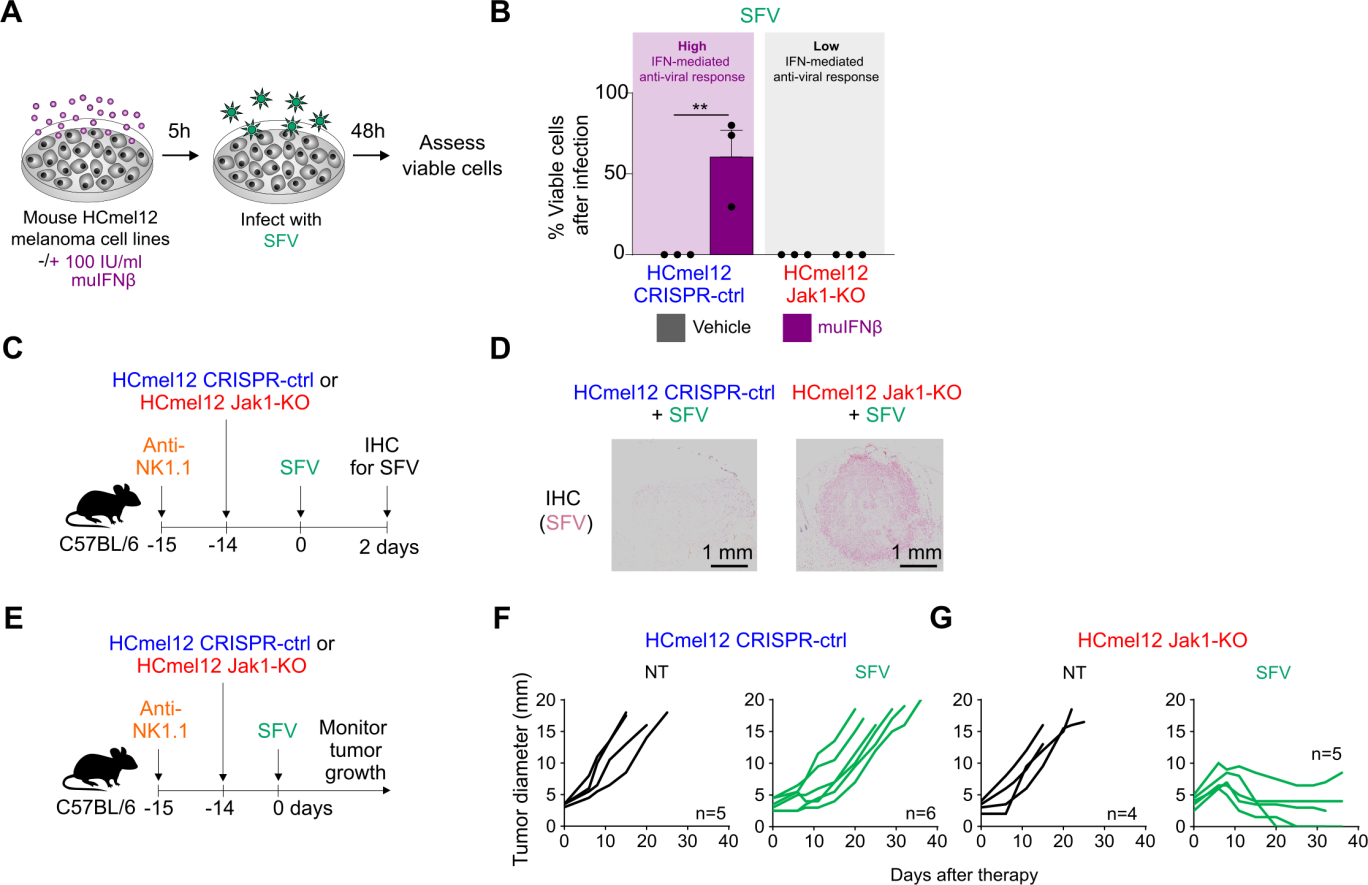
Genetically IFN signaling-deficient melanoma cell clones can be targeted by oncolytic virus *in vitro* and *in vivo.* **(A)** Graphical representation of experiment to assess impact of mouse IFNβ on infection with SFV and on viral oncolysis of the indicated HCmel12 melanoma cells. **(B)** Data from **(A)** represented as mean ± SEM and compared statistically with two-tailed unpaired t-test (**p<0.01). **(C)** Groups of 4–5 mice bearing established HCmel12 CRISPR-ctrl or HCmel12 Jak1-KO tumors were injected with 10^6^ PFU of SFV oncolytic virus, **(D)** Tumor tissue from **(C)** was harvested two days later, and stained for the presence of SFV by immunohistochemistry (IHC). **(E)** Groups of 4–5 mice bearing established HCmel12 CRISPR-ctrl or HCmel12 Jak1-KO tumors were injected with 10^6^ PFU of SFV oncolytic virus or left untreated and monitored for tumor growth over time. **(F, G)** Individual growth curves of mice from **(E)** bearing HCmel12 CRISPR-ctrl **(F)** or HCmel12 Jak1-KO tumors **(G)**.

Since IFN-unresponsive cells have diminished anti-viral defense, we hypothesized that oncolytic virotherapy could specifically eradicate immune evasive IFN-unresponsive melanoma cells that emerge following immunotherapy. Consistently, recombinant mouse IFNβ was able to protect HCmel12 CRISPR-ctrl, but not HCmel12 Jak1-KO cells, against lysis by oncolytic Semliki Forest Virus (SFV) as evidenced by crystal violet staining of surviving cells after 72 h (**Figures 4a, b**). We next sought to explore whether IFN-unresponsive HCmel12 Jak1-KO melanoma cells were also more susceptible to infection and lysis by SFV *in vivo*. Consistent and synchronous growth of transplanted HCmel12 Jak1-KO melanoma cells in syngeneic C57BL/6 mice required antibody-mediated depletion of NK cells prior to tumor injection (14), likely because these cells constitutively lack MHC-I expression (see **Figure 1b**). NK cell depleted wild-type mice bearing established IFN-responsive HCmel12 CRISPR-ctrl or IFN-unresponsive HCmel12 Jak1-KO cells were treated with a single intratumoral injection of SFV (**Figure 4c**). Tumor tissues were harvested two days later and stained for the presence of SFV using immunohistochemistry. Infection of melanoma cells by SFV *in vivo* could readily be detected in HCmel12 Jak1-KO tumors, but not in HCmel12 CRISPR-ctrl tumors (**Figure 4d**), consistent with the inability of SFV to efficiently infect IFN-responsive tumor cells *in vitro*. To assess the impact of oncolytic virotherapy on the *in vivo* tumor growth, we treated groups of NK cell depleted wild-type mice bearing established HCmel12 CRISPR-ctrl or HCmel12 Jak1-KO tumors with a single intratumoral injection of SFV (**Figure 4e**). Injections of SFV did not affect the growth of HCmel12 CRISPR-ctrl tumors (**Figure 4f**). In stark contrast, the growth of HCmel12 Jak1-KO tumors was abrogated following SFV injection (**Figure 4g**). Thus, genetically IFN-unresponsive melanoma cells are highly susceptible to viral oncolysis not only following treatment with recombinant IFN *in vitro* but also *in vivo*.

**Figure 5:**
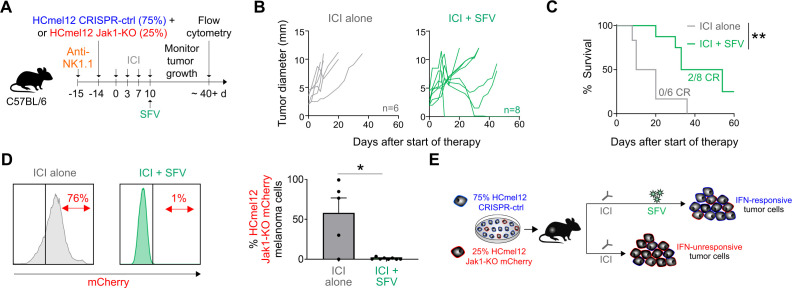
Genetically IFN signaling-deficient melanoma cell clones that emerge following ICI immunotherapy can be counter-selected with sequential oncolytic virotherapy **(A)** Groups of mice were inoculated with a mixture of HCmel12 CRISPR-ctrl (75%) and HCmel12 Jak1-KO-mCherry (25%) mouse melanomas and established tumors treated with dual immune checkpoint inhibitor therapy (ICI: anti-CTLA-4 + anti-PD-1) twice weekly for two weeks. 10 days after the start of therapy, mice were injected with 10^6^ PFU of SFV or left untreated. **(B)** Individual tumor growth curves in groups of mice treated as indicated. **(C)** Corresponding Kaplan-Meier survival graph (**p<0.005). **(D)** Representative histograms showing the percentage of HCmel12 Jak1-KO-mCherry cells in progressively growing tumors of the indicated treatment groups. Corresponding cumulative results of the experiment shown in **(A)**. Data represented as mean ± SEM and compared statistically with a two-tailed unpaired t-test (*p<0.05). **(E)** Graphical representation of the genetic evolution of melanoma cell subpopulations in response to the indicated therapeutic protocols.

We next hypothesized that oncolytic virotherapy could counteract IFN-unresponsive melanoma cells which emerge following immunotherapy. To experimentally investigate this hypothesis, we generated heterogeneous tumors by inoculating mixtures of 25% IFN-unresponsive HCmel12 Jak1-KO melanoma cells expressing mCherry and 75% IFN-responsive HCmel12 CRISPR-ctrl melanoma cells into 3 groups of NK cell-depleted wild-type mice (**Figure 5a**). When tumors reached a size of 3–5 mm in diameter (~14 days), two groups of mice received intraperitoneal injections with anti-PD-1 and anti-CTLA4 mAbs. One group received only ICI (“ICI alone”) and other the group received ICI with a subsequent intratumoral injection of SFV (“ICI + SFV”). Additional SFV injections in ICI-treated mice caused tumor regressions (**Figure 5b**). However, this effect was transient in the majority of mice and tumors still escaped immune control (**Figure 5c**).

Flow cytometric analysis of escaping tumors showed that ICI therapy selected for IFN-unresponsive HCmel12 Jak1-KO-mCherry cells, consistent with clinical observations. In contrast, sequential application of ICI therapy and oncolytic virotherapy selected for IFN-responsive HCmel12 CRISPR-ctrl melanoma cells (**Figure 5d**). This indicates that additional injections of SFV eradicated IFN-unresponsive HCmel12 Jak1-KO cell subpopulations. These experimental results demonstrate that oncolytic virotherapy can counteract the genetic selection of IFN-unresponsive tumor cell subpopulations that resist ICI therapy. However, progressively growing tumors consisting mostly of IFN-responsive HCmel12 CRISPR-ctrl melanoma cells were able to re-emerge in most mice. Therefore, sequential virotherapy counter-selects against IFN-signaling deficient subclones which emerge following immunotherapy (**Figure 5e**).

### The efficacy of combination immuno-virotherapy is abrogated by the emergence of IFN-responsive tumor cell subpopulations with a dedifferentiated phenotype

**Figure 6:**
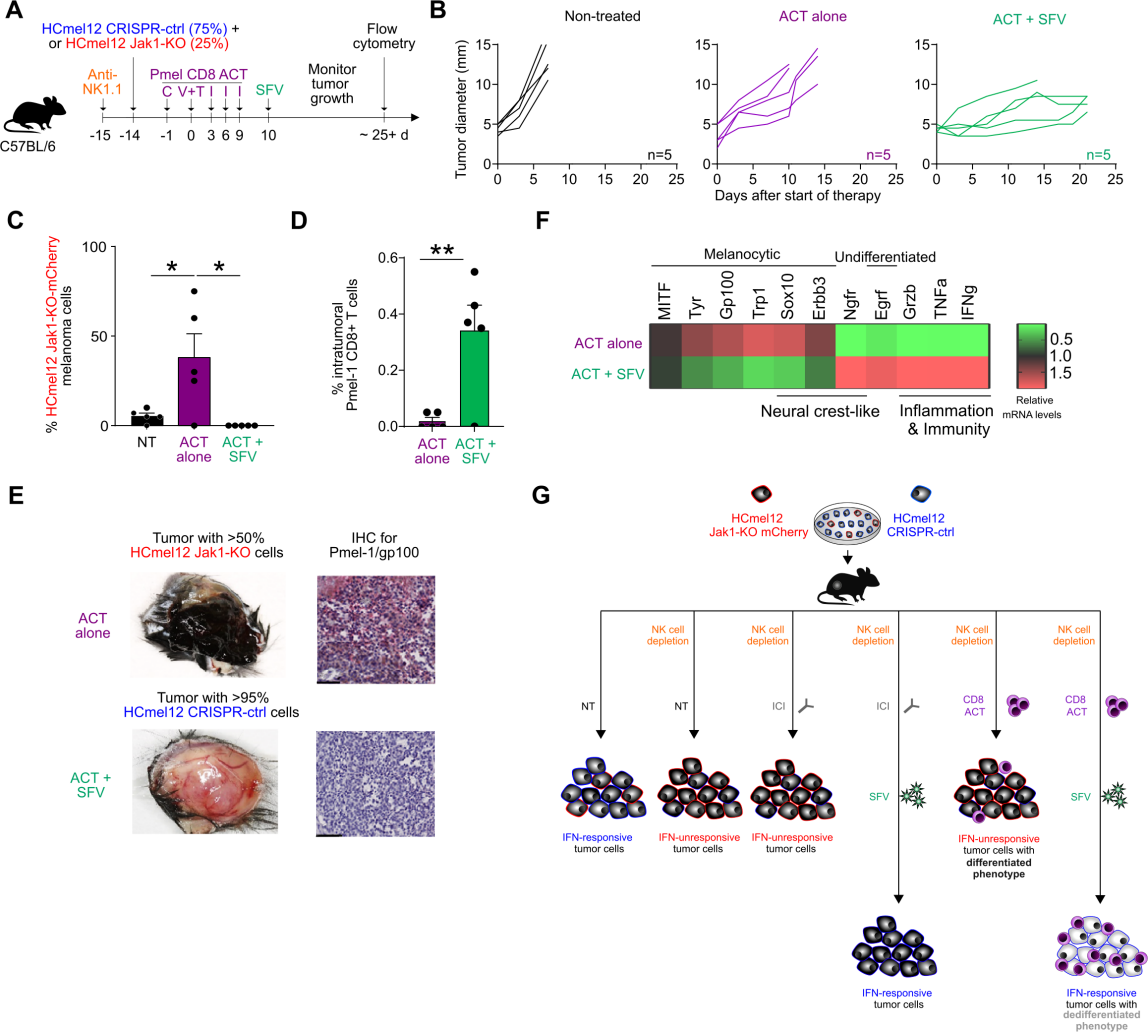
Adoptive T cell immunotherapy followed by oncolytic virotherapy promotes the re-emergence of IFN-responsive melanoma cell subpopulations with a dedifferentiated phenotype. **(A)** Groups of 4–5 mice were inoculated with a mixture of HCmel12 CRISPR-ctrl (75%) and HCmel12 Jak1-KO-mCherry (25%) mouse melanomas and established tumors treated with the “CVTI” CD8 ACT protocol (C, cyclophosphamide pretreatment; V, vaccination with recombinant Ad-gp100; T, adoptive transfer of TCRtg Pmel-1 CD8+ T cells; I, Injections of the innate stimuli polyI:C and CpG). 10 days after adoptive T cell transfer, mice were either injected with 10^6^ PFU of SFV or left untreated. **(B)** Individual tumor growth curves in groups of mice of a representative experiment that was repeated with similar results. **(C, D)** Percentage of HCmel12 Jak1-KO-mCherry cells **(C)** and of Pmel-1 CD8+ T cells **(D)** in progressively growing tumors of the indicated treatment groups. Data represented as mean ± SEM and compared statistically using a one-way ANOVA with Tukey *post hoc* (*p<0.05) or two-tailed unpaired t-test (**p<0.01). **(E)** Left: Representative macroscopic images of progressively growing tumors in the indicated treatment groups. Right: Corresponding microscope image of tumor tissue stained for expression of the T cell target antigen pmel-1/gp100. **(F)** Expression levels of gene sets for melanocytic, neural crest like, undifferentiated and inflamed melanoma cell phenotypes in the indicated treatment groups. Data represented as mean expression level in tumors obtained from the experiment shown in **(B)**. **(G)** Graphical summary of the genetic and phenotypic evolution of melanoma cell subpopulations in response to the indicated therapeutic protocols.

Phenotypic plasticity allows tumors to evade immune and viral pressures (20). Building on the observation that oncolytic virotherapy can eliminate IFN-unresponsive cells, we next investigated whether this selective pressure drives the emergence of dedifferentiated, IFN-responsive melanoma cells that escape CD8+ T cell targeting by downregulating the differentiation antigen gp100. Therefore, to further study the impact of sequential immuno-virotherapy on the dynamics of IFN-responsive and IFN-unresponsive melanoma cell subpopulations in heterogeneous tumors we employed an experimental adoptive cell transfer (ACT) therapy model established in our previous work (14, 19). This “CD8 ACT” protocol includes chemotherapeutic pre-conditioning with cyclophosphamide, adoptive transfer of Pmel-1 TCRtg CD8+ cytotoxic T cells together with adenoviral vaccination using recombinant Ad-gp100 that encodes the antigen recognized by Pmel-1 TCRtg CD8+ T cells, and adjuvant intratumoral injections of the immunostimulatory oligonucleotides polyI:C and CpG. We hypothesized that injections of SFV following CD8 ACT would enhance the effector functions of adoptively transferred, tumor-specific CD8+ T cells. Again, we inoculated three groups of NK cell-depleted wild-type mice with a mixture consisting of 25% IFN-unresponsive HCmel12 Jak1-KO-mCherry and 75% IFN-responsive HCmel12 CRISPR-ctrl melanoma cells. When tumors reached a size of 3–5 mm in diameter (~14 days), two groups of mice received CD8 ACT immunotherapy. One of these groups was subsequently injected intratumorally with SFV. The third group remained untreated (**Figure 6a**). CD8 ACT immunotherapy impaired tumor growth compared to controls resulting in prolonged tumor control. Additional intratumoral injections of SFV further delayed tumor progression but, unexpectedly, again did not cause significant tumor regressions (**Figure 6b**).

Flow cytometric analysis of progressively growing tumors confirmed that CD8 ACT therapy selected for IFN-unresponsive HCmel12 Jak1-KO-mCherry cells and that these tumor cell subpopulations were eradicated by additional injections of SFV (**Figure 6c**). Significantly increased numbers of adoptively transferred Pmel-1 CD8+ T cells were observed in tumors of mice that were injected with oncolytic virus, compared to tumors treated with CD8 ACT therapy alone, consistent with our hypothesis that oncolytic viruses promote CD8+ T cell effector functions in tumor tissues (**Figure 6d**). Strikingly, these tumors with residual Pmel-1 CD8+ T cells that consisted predominantly of IFN-responsive HCmel12 CRISPR-ctrl cells, exhibited amelanotic phenotypes (**Figure 6e**) and strongly downregulated the expression of the CD8 ACT target antigen Pmel-1/gp100. In contrast, tumors of mice treated with only CD8 ACT that consisted mostly of IFN-unresponsive HCmel12 Jak1-KO-mCherry cells, appeared darkly pigmented (**Figure 6e**) and retained expression of Pmel-1/gp100. Gene expression analyses using quantitative RT-PCR confirmed strong expression of the microphthalmia-associated transcription factor (MITF)-driven melanocytic gene program in pigmented tumors. This program was down-regulated in amelanotic tumors that strongly expressed inflammatory genes, as well as nerve growth factor receptor (Ngfr), known to be expressed in melanocytic progenitor cells (**Figure 6f**). Taken together, these experimental observations indicated that our CD8+ T cell-directed immunotherapy, that targets a differentiation antigen, rapidly selects for IFN-unresponsive melanoma cell subpopulations with a differentiated phenotype. Additional injections of oncolytic viruses eliminate IFN-unresponsive melanoma cells, reactivate intratumoral CD8+ T cells, and enforce a phenotypic shift of the residual IFN-responsive melanoma cells towards dedifferentiated, neural crest-like cell states in an inflammatory environment that resists both immuno- and virotherapy (**Figure 6g**).

## Discussion

Our findings experimentally demonstrate that oncolytic virotherapy can effectively target and eliminate IFN-unresponsive tumor cell subclones that emerge following ICI and ACT immunotherapy *in vivo*. Unexpectedly, we observed that IFN-responsive tumor cells re-emerged with a dedifferentiated phenotype that resisted both T cell control and viral oncolysis. Together, our findings highlight dedifferentiation as a potential convergent resistance mechanism that ultimately limits the efficacy of both treatment approaches.

We initially demonstrate that HCmel12 Jak1-KO melanoma cells, lacking key components of both type I and II IFN signaling, fail to upregulate MHC-I in response to IFN stimulation, rendering them susceptible to NK cell-mediated killing *in vitro*. In immunocompetent mice, these IFN-unresponsive, MHC-deficient tumors rarely establish. However, these IFN signaling-deficient tumors grew robustly upon NK cell depletion, underscoring the importance of innate immune surveillance in controlling IFN-deficient tumors (21). In NK cell-depleted mice bearing mixed populations of IFN-responsive and IFN-unresponsive tumor cells, treatment with ICI immunotherapy led to the selective outgrowth of the IFN-unresponsive subclones, in agreement with a previously published observation in a mouse melanoma model (22) and the observation in patient care that tumor cell variants with genetic loss of IFN signaling pathway components are associated with ICI therapy resistance (9, 10).

Loss of IFN signaling in tumor cells not only facilitates immune evasion, but also creates a vulnerability to viral oncolysis. This paradox highlights the need to strategically apply oncolytic viruses and tailor their use to the IFN landscape of individual tumors. Importantly, we identify oncolytic virotherapy as a selective therapeutic strategy to target IFN-unresponsive melanoma subpopulations that emerge following immunotherapy. Both *in vitro* and *in vivo*, IFN-unresponsive tumor cells were highly susceptible to SFV-mediated lysis, whereas IFN-responsive melanoma cells were protected, likely by their intact anti-viral response. This agrees with observations that inhibition of IFN-signaling components enhances the abilities of oncolytic virotherapy to control tumors (12, 23, 24). When used sequentially following ICI therapy, SFV selected against the emergence IFN-unresponsive HCmel12 Jak1-KO cells in heterogeneous tumors. These findings suggest that deficiencies in the IFN-signaling pathway provide a route for immune escape and simultaneously creates a unique therapeutic vulnerability to oncolytic viruses.

In our experiments, elimination of immunotherapy-resistant, IFN-unresponsive clones through subsequent oncolytic virotherapy did not eradicate all routes of resistance. Instead, tumor cells with a dedifferentiated phenotype emerged that resists both immune and viral attack. CD8 ACT immunotherapy targeting the melanocyte differentiation antigen gp100 in mice bearing heterogeneous tumors led to outgrowth of IFN-unresponsive, pigmented melanoma subpopulations that retained antigen expression. Sequential treatment with SFV ablated these IFN-unresponsive subclones. However, tumors continued to grow and were composed predominantly of IFN-responsive, dedifferentiated melanoma cells that had lost pigmentation and downregulated gp100 expression. This phenotypic plasticity represents an adaptive response of tumor cells, where immune and viral pressures drive dedifferentiation toward a neural crest-like state that is associated with immune resistance (20). This is consistent with recent work showing that dedifferentiated melanoma cell states are linked to resistance to ICI, including neural crest stem cell-like states associated with immune exclusion and tumor cell programs that promote T cell exclusion under therapeutic pressure (25–27). This is reminiscent of how stem cells protect themselves from viral destruction by intrinsically increasing expression levels of IFN-stimulated genes (28). We postulate that cancer cells may exploit this process to resist oncolytic virotherapy, since a subset of interferon-stimulated genes (ISGs) with known anti-viral functions not only block viral replication (29), but also drive resistance to multiple treatment modalities (30) including immunotherapy and virotherapy.

Our findings touch upon the paradoxical anti- and pro-tumoral roles of IFN-signaling in cancer cells that have been described in the literature (31, 32). Initial CRISPR-based genetic screens *in vivo* reported that defects in tumor IFN signaling confer cancer cell-intrinsic resistance to T cell killing (8). In contrast, subsequent CRISPR-based genetic screens *in vivo* revealed that loss of tumor IFNg signaling can sensitize multiple cancer models to immune attack (33). This touches upon the ongoing debate over the importance of genetic selection for IFN-unresponsive clones in melanoma patients following immunotherapy. Song et al. (34) compiled functional genomic data from 33 CRISPR screens and demonstrated that while loss of function mutations in IFNg signaling pathways were consistently enriched in cancer cells that resisted T cell killing *in vitro*, these mutations were frequently counter-selected in immunocompetent mice and following ICI immunotherapy *in vivo*. In support of the unexpected experimental evidence for a tumor cell-protective role of IFNg signaling under immunotherapy in mice *in vivo*, their meta-analysis of over 2, 000 ICI-treated patients revealed an association between loss of function mutations in IFNg signaling pathways and improved clinical responses to ICI therapy. Future investigations will have to elucidate the circumstances that determine the evolutionary dynamics of IFNg signaling in cancer cells.

In our model, the paradoxical anti- and pro-tumoral roles of tumor-cell intrinsic IFN signaling map directly onto the resistance phenotypes observed during sequential therapy. Loss of IFN signaling promotes escape from T cell-directed immunotherapy, consistent with the impaired MHC-I upregulation and selective outgrowth of IFN-unresponsive Jak1-KO subclones under both ICI and ACT, but at the same time creates a vulnerability to NK cell surveillance and SFV-mediated viral oncolysis. By contrast, the tumor cells that re-emerged after sequential immuno-virotherapy were IFN-responsive and displayed a dedifferentiated phenotype, indicating that intact IFN signaling may support an anti-viral, inflammatory-resistant state that protects residual cells from viral control while phenotypic plasticity reduces susceptibility to antigen-directed T cells. Thus, the present study suggests that sequential immuno-virotherapy does not simply eliminate resistance, but rather selects for two distinct escape states under different therapeutic pressures: an IFN-unresponsive, differentiated immune-evasive state under immunotherapy alone, and an IFN-responsive, dedifferentiated escape state following subsequent oncolytic virotherapy.

Our experimental findings may offer an explanation why the phase III clinical trial that combined immunotherapy with antibodies blocking PD1 and oncolytic virotherapy with Talimogene laherparepvec (TVEC) did not meet clinical expectations (35), despite encouraging early results in phase I (5). They also provide a rationale for adding JAK inhibitors targeting chronic IFN signaling (36) into combination immuno-virotherapy strategies to overcome IFN-driven resistance. This notion is supported by recent clinical trials which have demonstrated that combining JAK inhibitors with ICI can reinvigorate exhausted T cells and reshape the tumor immune microenvironment, thereby enhancing immunotherapy efficacy in patients with refractory cancers such as Hodgkin lymphoma (37) and non-small cell lung cancer (38). In line with these clinical results, a recent study revealed an association between the presence of neutralizing autoantibodies against type I IFNs in the serum and responses to ICI in patients (39). These findings support the notion that neutralization of type I IFNs or blockade of IFN signaling may paradoxically boost anti-tumor immunity, offering a potential strategy to overcome resistance driven by prolonged IFN pathway activation in dedifferentiated cancer cells.

Previous studies have shown that oncolytic virotherapy can enhance intratumoral immune responses and improve the activity of checkpoint blockade through dynamic immune modulation and T cell priming, while antiviral signaling can simultaneously restrict viral replication and spread (40–42). This is consistent with our observation that SFV not only eliminated IFN-unresponsive tumor cells but also increased the number of intratumoral Pmel-1 CD8+ T cells following adoptive T cell therapy. These observations are also in line with recent work highlighting the capacity of oncolytic virotherapy to remodel the immunosuppressive tumor microenvironment and to act in multi-synergistic combination strategies (43, 44). Building on this, our findings suggest that the efficacy of combined immunotherapy and oncolytic virotherapy may be limited by a shift between distinct tumor escape routes. While sequential immuno−virotherapy can counter−select IFN−unresponsive melanoma subclones that emerge under immune pressure, tumor cells can subsequently re−emerge in an IFN−responsive, dedifferentiated, neural crest−like state that resists both T cell control and viral oncolysis. In our model, this highlights phenotypic plasticity as a convergent resistance mechanism that ultimately limits sustained therapy responses. These observations argue for optimizing sequential immuno−virotherapy strategies, including treatment timing and scheduling, transient modulation of JAK/IFN signaling, and multimodal approaches that also target dedifferentiated melanoma cell states. These concepts remain speculative in the context of our study but provide a framework for future translational development.

Our results provide important insights into the evolutionary dynamics of IFN-dependent resistance in sequential immuno−virotherapy. However, several aspects of this study should be interpreted with caution. First, our findings are based on the HCmel12 melanoma model, and it remains to be determined to what extent these results apply to other tumor models and genetic backgrounds. Second, we focused on a single oncolytic virus (SFV-VA7), and whether similar evolutionary dynamics occur with other viruses remains unclear. Finally, experiments were performed in male mice, and potential sex-specific differences in immune responses and therapy outcomes were not addressed. These limitations should be considered when extrapolating our findings and underscore the need for validation in additional models.

Collectively, these findings reveal the dynamic evolution of IFN-unresponsive subclones under immune pressure and show that subsequent oncolytic virotherapy can temporarily counter-act immune resistant IFN signaling deficient clones that emerge following immunotherapy. However, the emergence of dedifferentiated cancer cells, shaped by convergent resistance mechanisms, ultimately limits the sustained effectiveness of this sequential immuno-virotherapy strategy. Effective long-term tumor control may require the strategic combination of therapies that not only eradicate immune-evasive subclones but also prevent or re-target the emergence of dedifferentiated, therapy-resistant tumor phenotypes.

## Data Availability

The datasets presented in this study can be found in online repositories. The names of the repository/repositories and accession number(s) can be found in the article/**Supplementary Material**.
